# Short-Term Effects of Sibutramine on Mineral Status and Selected Biochemical Parameters in Obese Women

**DOI:** 10.1007/s12011-012-9425-6

**Published:** 2012-04-27

**Authors:** Joanna Suliburska, Paweł Bogdański, Monika Szulińska, Danuta Pupek-Musialik

**Affiliations:** 1 Department of Human Nutrition and Hygiene, Poznan University of Life Sciences, Wojska Polskiego 31 Str., 60-624 Poznan, Poland; 2 Department of Internal Medicine, Metabolic Disorders and Hypertension, Poznan University of Medical Sciences, Szamarzewskiego 84 Str., 60-569 Poznan, Poland

**Keywords:** Sibutramine, Minerals, Lipid profile, Glucose, Obesity, Women

## Abstract

The aim of this study was to assess the effect of sibutramine on mineral status and selected biochemical parameters in obese women. The study was conducted on 24 patients who received 15 mg daily doses of sibutramine for 12 weeks, and on 20 patients who received placebo. At the baseline, after the sixth and twelfth weeks of treatment, body weight and blood pressure were measured, the BMI was calculated, and samples of blood and of first morning urine were collected. Serum lipid profiles, glucose levels, and nitric oxide levels were determined. The iron (Fe), copper (Cu), zinc (Zn), calcium (Ca), and magnesium (Mg) present in the serum and urine samples were assessed. The erythrocyte hemolysate of the patients was use to assay the activity of glutathione peroxidase (GSH-Px) and superoxide dismutase (SOD). No changes were observed in BMI, blood pressure, or nitric oxide during the study. After 12 weeks of treatment, a decrease was observed in total cholesterol, LDL cholesterol, triglyceride, glucose, and ferritin levels. GSH-Px and SOD activity increased after 12 weeks of sibutramine treatment. The Mg and Cu increases was observed in serum after the sixth and twelfth weeks of treatment. It was found that the Zn level decreased in serum after the twelfth week. The elimination of Ca, Mg, Fe, Zn, and Cu in urine also declined in the twelfth week. No differences were found in the women taking the placebo. In conclusion, we found that sibutramine had a positive effect on lipid and glucose status in obese women. However, the drug disturbed the balance of minerals, especially Zn and Mg, in the subjects.

## Introduction

The prevalence of obesity and its comorbidities has been increasing all over the world. Abdominal or visceral obesity is closely related to disturbances in lipid and glucose status, and also to a lower antioxidative status in the body [1]. Weight loss reduces the cardiovascular risks associated with obesity [2]. The treatment of obesity should be individually tailored and should be maintained for a long term. The first line of strategy for weight loss is a combination of diet, physical activity, and behavior modification. Anti-obesity drugs may be used in adult patients when dietary and lifestyle modifications have been unsuccessful.

For a long time, sibutramine was used as an effective anti-obesity drug in Europe, but because of the cardiovascular risk associated with it, European regulators suspended its market authorization, and the US Food and Drug Administration restricted its license. This study was conducted a few months before the withdrawal of sibutramine’s license. Over the years, the effectiveness and side effects of sibutramine have been assessed in many studies. Subsequent studies have shown that the drug has a significant effect on weight loss, due to its satietogenic and calorigenic effects [3]. It also improved glycemic and lipid profile and uric acid, and for this reason, it was often recommended in diet and lifestyle advice [4]. Svacina et al. [5] documented the positive effect of a 3-month sibutramine treatment on dyslipidemia in the obese, even in those already being treated with statins. In studies with hypertensive subjects whose blood pressure was adequately controlled by antihypertensive drugs, a slight to significant decrease in systolic and diastolic blood pressure was observed with sibutramine use [6]. Moreover, Derosa et al. [7] found that sibutramine plus L-carnitine gave a greater improvement in body weight loss and glycemic and lipid profile compared to sibutramine alone. Sibutramine therapy has been associated with significant C-reactive protein reduction as compared with routine treatment in patients with coronary artery disease. Thus, short-term (4 months) therapy with sibutramine, together with diet and lifestyle modifications, was associated with improved endothelial function [8] but did not affect TNF-alpha [9]. In our previous unpublished research, we observed a considerable decrease in ferritin and Zn concentration in the serum of patient undergoing treatment with sibutramine. A review of the literature on the topic showed that there was limited data on the effects of sibutramine on mineral balance in obese women. The aim of this study was thus to assess the effect of sibutramine on mineral status and on selected biochemical parameters in obese women.

## Material and Methods

### Study Design

The study was conducted at the Department of Internal Diseases, Metabolic Disorders, and Arterial Hypertension, Poznan University of Medical Sciences (Poland), and at the Department of Human Nutrition and Hygiene, Poznan University of Life Sciences (Poland). The study protocol was approved by Bioethics Commission at Poznan University of Medical Sciences (approval no. 86/09).

### Patients

In the Clinic of the Department of Internal Diseases, Metabolic Disorders, and Hypertension in Poznan, we treated obese patients with sibutramine. During the biochemical studies carried out in women taking sibutramine, we observed a considerable decrease in ferritin and Zn concentration in serum. In addition, these women often complained about the brittleness of their nails and hair and of taste disorders, which may have been due to low concentrations of Zn in their bodies. This imbalance of mineral status, apparently due to the use of sibutramine, was disturbing, especially when it is known that an appropriate level of minerals, particularly of Ca and Fe, is very important for the health of women of childbearing age. We thus decided to conduct a study accessing the effect of sibutramine on mineral status in obese women.

The study was conducted on 44 obese women (body mass index [BMI] ≥ 30 kg/m^2^) aged 40.6 ± 12.1 years. Individuals suffering from chronic disease or using any medications (including OTC products, with the exception of five women who took oral contraceptives) were excluded. For at least 3 months before the study, all women have remained on a controlled-energy diet (with nearly 500 kcal daily deficit) that took 58 % of its calories from carbohydrates, 27 % from fat, and 15 % from proteins, with a maximum cholesterol content of 300 mg/day and 35 g/day of fiber. Standard dietary advice was given by a qualified dietitian. Subjects did not change their diet during the study and were asked to restrain from smoking for the time of the study.

Women who were pregnant, breastfeeding, or capable of becoming pregnant were excluded. All subjects were informed about the study’s purpose, protocol, and risks. All individuals provided informed written consent.

### Treatment and Assessment

Twenty-four patients aged 40.6 ± 12.1 were assigned to receive sibutramine 15 mg once daily for 12 weeks, while 20 patients aged 40.7 ± 12.2 received placebos containing pure microcrystalline cellulose. Before the beginning of the study, all patients underwent a full physical examination. Anthropometric measurements of the subjects wearing light clothing and no shoes were conducted. Weight was measured to the nearest 0.1 kg, and height to the nearest 0.1 cm. The BMI was calculated by dividing the weight (kg) by the height squared (m^2^). Obesity was defined as BMI ≥ 30 kg/m^2^. Blood pressure was measured according to the guidelines of the European Society of Hypertension using a digital electronic tensiometer (model 705IT, Omron Corporation, Kyoto, Japan). Regular or large adult cuffs were used, depending on the patient’s arm circumference. The measurements were taken while fasting in the morning hours in a sitting position, with the legs uncrossed, the back and arms supported. Hypertension was defined by measurement of arterial blood pressure as the average of three measurements obtained after 10 min of physical resting (3 times at 2 different visits).

All participants had blood collected from a forearm vein in serum-separated tubes (without using an anticoagulant). The coagulated blood was left to clot at room temperature for 30 min, and then centrifuged for 15 min at 2,000 rpm at 4°C. Then the supernatant fluid was separated. Serum samples were stored at −20°C for no longer than 2–3 days. Blood samples were collected after an overnight fast, and after 30 min in the supine position.

Measurements of total cholesterol (TC), high density lipoproteins (HDL-C), low density lipoproteins (LDL-C), triglycerides (TG), and glucose were performed on each blood sample. Prior to the Fe, Cu, and Zn serum concentration assays, the serum was diluted with triton X-100 (Merck), and before the Ca and Mg concentration assays, the serum was diluted with triton and LaCl_3_ (Merck).

Urine was collected in the morning, following an overnight fast. Morning urine samples were collected in sterilized vessels and stored for material analysis.

The samples of urine were mineralized in a mixture of concentrated nitric (65 %) and perchloric (60 %) acids (suprapure, Merck; *v*/*v*, 1:1) in a microwave oven (Milestone), and dissolved (*v*/*v*, 1:1) in Triton X-100 (Merck; all minerals) and LaCl_3_ (Merck; Ca and Mg).

After the sixth and twelfth weeks of treatment, weight and blood pressure were measured, BMI was calculated, and the blood samples and first morning urine were collected from all patients. All serum and urine parameters were determined after a 12-h overnight fast.

### Biochemical Assays

The content of Fe, Cu, Zn, Ca, and Mg in the serum and urine samples was determined by the flame atomic absorption spectrometry (AAS-3 spectrometer, Carl Zeiss, Germany with deuterium background correction). In order to obtain concentrations of the serum bioelements, the samples were diluted (*v*/*v*, 1:1) as follows: for Fe, Zn, and Cu analyses 0.01 % Triton X-100 (Merck) was used, while for the Mg and Ca analysis, aqueous solutions consisting of 0.01 % Triton X-100 (Merck) and 0.05 % lanthanum chloride (Merck) were used. The content of Fe, Cu, Zn, Ca, and Mg in serum and urine samples was determined at the following wavelengths: 248.3 nm (Fe), 324.8 nm (Cu), 213.9 nm (Zn), 422.7 nm (Ca), and 285.2 nm (Mg).

The accuracy of the method was verified using certified reference materials (HUM ASY CONTROL 2 and URN ASY CONTROL 2, Randox), and reached 95 %, 99 %, 94 %, 99 %, and 102 % for Ca, Mg, Fe, Zn, and Cu, respectively [10].

Plasma TC, LDL-C, HDL-C, TG, and glucose levels were measured using commercial kits. The accuracy and precision of the techniques used to assay the lipids and glucose were validated. Reproducibility was checked with a human serum control (Randox). Accuracy was assessed by means of the recovery value and its range between 95 % and 109 %, and the variability coefficient (an indicator of the method’s precision) did not exceed 10 %.

The activity of glutathione peroxidase (GSH-Px) was assayed in the erythrocyte hemolysate using a method of Paglia and Valentine [11]. GSH-Px decomposes hydrogen peroxide, causing oxidation of the reduced glutathione. Oxidized glutathione is then reduced in a reaction that is catalyzed by glutathione reductase. The coenzyme of the reaction is reduced nicotinamide adenine dinucleotide phosphate, which is transformed into oxidized form, and causes a change in light absorbance at 340 nm (spectrophotometer Sunrise, Tecan). Superoxide dismutase (SOD) activity was determined in the erythrocyte hemolysate using a modification of epinephrine-adenochrome detection system [12]. The enzyme inhibits the reaction of adrenaline autooxidation to adenochrome in an alkaline environment. The unit of SOD activity under the given conditions is defined as the amount of enzyme that inhibits 50 % of the reaction at the maximal increase of absorbance rectilinear segment of adenochrome formation at wavelength 450 nm (spectrophotometer Sunrise, Tecan). Erythrocyte GSH-Px and SOD activity was determined using a commercial kit (Randox), and expressed as units per gram of hemoglobin. The hemoglobin concentration in milligrams per milliliters was determined using the cyanmethemoglobin method [13].

Erythrocyte lysate was prepared from blood collected with EDTA. Blood was centrifuged at 700–1,000×*g* at 4°C. Plasma was then removed. The red cells were washed three times with cold isotonic saline solution. Erythrocytes were lysed with a ninefold volume of ice-cold HPLC-grade water, and then centrifuged at 10,000×*g* at 4°C. Supernatant was used for assaying.

The concentration of ferritin in the serum was determined by a two-site sandwich immunoassay using direct chemiluminometric technology (ADVIA Centaur XP analyzer), which uses constant amounts of two anti-ferritin antibodies [14].

The concentration of nitric oxide (NO) was determined using the spectrophotometric method on the serum, with the testing set by Oxis. The test determines nitric oxide concentration based on the enzymatic conversion of nitrate to nitrite by nitrate reductase. The reaction is followed by colorimetric detection of nitrate as an azo dye product of the Griess Reaction. The Griess reaction transforms nitrites to NO, which then reacts with sulfanilic acid, and the indirect product of the reaction interacts with N-(1-naphtyl) ethylendiamine, leading to the formation of a red-violet colored product having maximum absorbance at *λ* = 540 nm. The solution color change, measured spectrophotometrically (with Hyperion Micro Reader), is proportional to the nitric oxide concentration [15].

### Statistical Analysis

The experimental results were given as means ± standard deviation. The statistical analysis was carried out using STATISTICA 7.0 software and the ANOVA test. A *p* value less than 0.05 was considered statistically significant.

## Results

### Body Mass Index and Blood Pressure

The results of BMI and blood pressure measurements are presented in Table 1. No significant change was observed in BMI or in the systolic or diastolic blood pressure of patients during the study. However, the body mass index slightly decreased after 6 weeks (38.43 vs. 37.93, *p* = 0.345) and 12 weeks (38.43 vs. 36.83, *p* = 0.204). Both systolic and diastolic blood pressure did not change at all in comparison with the baseline.

**Table 1:** Baseline values

	Baseline		6 weeks		12 weeks	
	Sibutramine	Placebo	Sibutramine	Placebo	Sibutramine	Placebo
	(*N* = 24)	(*N* = 20)	(*N* = 24)	(*N* = 20)	(*N* = 24)	(*N* = 20)
N	24	20	–	–	–	–
Sex [F]	24	20	–	–	–	–
Age [years]	40.61 ± 12.13	40.72 ± 12.22	–	–	–	–
Sibutramine [dose]	15 mg	–	15 mg	–	15 mg	–
BMI [kg/m^2^]	38.43 ± 8.54	38.03 ± 5.76	37.93 ± 7.54	37.55 ± 6.21	36.83 ± 7.35	37.31 ± 4.83
SBP [mmHg]	133.52 ± 10.44	131.82 ± 8.37	133.18 ± 12.42	132.87 ± 8.91	133.57 ± 9.74	132.38 ± 8.31
DBP [mmHg]	85.53 ± 5.22	85.77 ± 5.42	85.68 ± 5.61	85.73 ± 5.34	85.42 ± 4.81	85.74 ± 4.11

Blood pressure and BMI changes at 6 and 12 weeks in the group during the study. Data are means ± SD

*mean* the arithmetic mean, *SD* standard deviation, *N* number of subjects, *BMI* body mass index, *SBP* systolic blood pressure, *DBP* diastolic blood pressure

### Biochemical Parameters

After 6 weeks of sibutramine treatment, a significant decrease was found only in TC (*p* = 0.031; Table 2). No change in LDL-C, TG, glucose, or ferritin levels was observed at 6 weeks. After 12 weeks, a significant decrease was observed for TC (*p* = 0.013), LDL-C (*p* = 0.034), TG (*p* = 0.044), glucose (*p* = 0.046), and ferritin (*p* = 0.030). GSH-PX and SOD activity markedly increased (*p* = 0.036 and *p* = 0.021, respectively) after 12 weeks of sibutramine treatment. Serum levels of NO did not change during the study. In women taking the placebo, no significant difference was observed.

**Table 2:** Biochemical parameters in serum changes at 6 and 12 weeks during the study

	Baseline		6 weeks		12 weeks	
	Sibutramine	Placebo	Sibutramine	Placebo	Sibutramine	Placebo
	(*N* = 24)	(*N* = 20)	(*N* = 24)	(*N* = 20)	(*N* = 24)	(*N* = 20)
TC [mmol/l]	4.41 ± 0.58	4.40 ±0.51	4.11 ± 0.57^a^	4.38 ± 0.56	4.08 ± 0.58^a^	4.35 ± 0.52
LDL-C [mmol/l]	2.56 ± 0.48	2.55 ± 0.51	2.35 ± 0.46	2.52 ± 0.49	2.28 ± 0.42^a^	2.50 ± 0.43
HDL-C [mmol/l]	1.25 ± 0.24	1.28 ± 0.21	1.26 ± 0.25	1.28 ± 0.23	1.25 ± 0.26	1.26 ± 0.21
TG [mmol/l]	1.37 ± 0.37	1.35 ± 0.38	1.21 ± 0.36	1.30 ± 0.30	1.15 ± 0.35^a^	1.30 ± 0.31
Glucose [mmol/l]	5.35 ± 0.47	5.31 ± 0.48	5.33 ± 0.46	5.30 ± 0.47	5.18 ± 0.27^a^	5.30 ± 0.50
Ferritin [μg/l]	91.12 ± 40.42	91.25 ± 41.22	78.15 ± 41.39	88.11 ± 40.08	65.51 ± 38.50^a^	85.14 ± 30.83
Nitric oxide (NO) [μmol/l]	10.60 ± 3.01	10.67 ± 2.67	10.83 ± 3.08	10.57 ± 2.68	10.67 ± 2.99	10.60 ± 2.68
Glutathione peroxidase [U/gHb]	15.62 ± 6.30	15.68 ± 7.11	18.26 ± 8.23	16.15 ± 7.83	19.52 ± 8.45^a^	16.22 ± 6.28
Superoxide dismutase [U/gHb]	2,570 ± 692	2,582 ± 5.82	2,853 ± 576	2,637 ± 583	3,554 ± 723^a^	2,628 ± 683

Data are means ± SD

*mean* the arithmetic mean, *SD* standard deviation, *N* number of subjects, *TC* total cholesterol, *LDL-C* LDL cholesterol, *HDL-C* HDL cholesterol, *TG* triglycerides

^a^Sibutramine group *p* < 0.05 vs baseline

### Minerals

A significant increase in Mg and Cu concentration was observed in the serum of women with sibutramine after 6 (*p* = 0.037 and *p* = 0.029, respectively) and 12 weeks (*p* = 0.030 and *p* = 0.021, respectively; Table 3). It was also found that Zn levels markedly decreased (*p* = 0.018) in serum after 12 weeks, and a slight decrease was also observed after 6 weeks of treatment. In the case of Fe serum concentration, a slight decrease was found during the study.

**Table 3:** Minerals in serum and urine changes at 6 and 12 weeks during the study

	Baseline		6 weeks		12 weeks	
	Sibutramine	Placebo	Sibutramine	Placebo	Sibutramine	Placebo
	(*N* = 24)	(*N* = 20)	(*N* = 24)	(*N* = 20)	(*N* = 24)	(*N* = 20)
Serum						
Ca [mmol]l]	2.54 ± 0.13	2.52 ± 0.12	2.65 ± 0.42	2.60 ± 0.21	2.52 ± 0.25	2.50 ± 0.33
Mg [mmol/l]	0.80 ± 0.07	0.83 ± 0.07	0.93 ± 0.06^a^	0.85 ± 0.06	0.95 ± 0.06^a^	0.88 ± 0.25
Fe [μmol/l]	17.85 ± 5.32	17.33 ± 5.18	16.82 ± 6.24	16.84 ± 5.10	16.35 ± 5.92	16.58 ± 5.03
Zn [μmol/l]	12.42 ± 2.42	12.04 ± 2.80	11.48 ± 2.38	11.96 ± 2.81	10.51 ± 2.58^a^	11.46 ± 2.91
Cu [μmol/l]	13.61 ± 1.81	13.03 ± 1.71	14.94 ± 1.78^a^	13.41 ± 1.80	15.68 ± 1.92^a^	13.63 ± 1.21
Urine						
Ca [mmol/l]	3.84 ± 2.53	3.21 ± 2.31	2.97 ± 2.09^a^	3.25 ± 2.30	2.89 ± 2.35^a^	3.18 ± 2.18
Mg [mmol/l]	5.84 ± 2.42	5.50 ± 2.80	3.25 ± 1.78^a^	5.00 ± 1.80	3.05 ± 1.50^a^	4.58 ± 2.91
Fe [μmol/l]	9.27 ± 5.21	9.30 ± 4.77	6.02 ± 4.20^a^	8.50 ± 4.18	5.78 ± 3.84^a^	7.90 ± 5.10
Zn [μmol/l]	9.38 ± 3.58	9.40 ± 3.37	8.46 ± 4.21	8.98 ± 4.80	7.78 ± 3.80^a^	9.12 ± 2.83
Cu [μmol/l]	1.02 ± 0.51	1.11 ± 0.68	0.68 ± 0.34^a^	0.98 ± 0.80	0.61 ± 0.35^a^	1.08 ± 0.56

Data are means ± SD

*mean* the arithmetic mean, *SD* standard deviation, *N* number of subjects

^a^Sibutramine group *p* < 0.05 vs baseline

The elimination of Ca, Mg, Fe, and Cu in urine declined significantly at 6 weeks (*p* = 0.032, *p* = 0.048, *p* = 0.037, and *p* = 0.026, respectively) and 12 weeks (*p* = 0.028, *p* = 0.031, *p* = 0.023, and *p* = 0.018, respectively) of the study (Table 3). The considerable decrease in Zn concentration in urine was observed only after 12 weeks (*p* = 0.017), but a slight decline in Zn elimination was evident after 6 weeks of the study.

It was found that the Ca/Mg and Zn/Cu molar ratios in serum significantly decreased at the sixth (*p* = 0.042 and *p* = 0.032) and twelfth weeks (*p* = 0.034 and *p* = 0.021) of the study (Table 4). A marked Cu/Fe molar ratio increase was present at 6 (*p* = 0.035) and 12 weeks (*p* = 0.028) vs. the baseline, and at 12 weeks vs. 6 weeks (*p* = 0.046). In urine, significant increases in the molar ratios of Ca/Mg and Zn/Cu, as well as a significant decrease in the Fe/Zn molar ratio at 6 (*p* = 0.016, *p* = 0.027, and *p* = 0.041, respectively) and 12 weeks (*p* = 0.013, *p* = 0.022, and *p* = 0.037, respectively) of sibutramine treatment were observed. No marked differences in women receiving the placebo were found.

**Table 4:** Mineral molar ratio in serum and urine changes at 6 and 12 weeks during the study

	Baseline		6 weeks		12 weeks	
	Sibutramine	Placebo	Sibutramine	Placebo	Sibutramine	Placebo
	(*N* = 24)	(*N* = 20)	(*N* = 24)	(*N* = 20)	(*N* = 24)	(*N* = 20)
Serum						
Ca/Mg	3.16 ± 0.21	3.04 ± 0.20	2.85 ± 0.27^a^	3.06 ± 0.25	2.66 ± 0.23^a^	2.84 ± 0.20
Fe/Zn	1.44 ± 0.12	1.44 0 ± .13	1.47 ± 0.12	1.41 ± 0.14	1.56 ± 0.12	1.45 ± 0.10
Cu/Fe	0.76 ± 0.09	0.75 ± 0.10	0.89 ± 0.05^a^	0.80 ± 0.04	0.96 ± 0.05^a,b^	0.82 ± 0.07
Zn/Cu	0.92 ± 0.11	0.92 ± 0.12	0.77 ± 0.12^a^	0.89 ± 0.18	0.66 ± 0.12^a^	0.86 ± 0.09
Urine						
Ca/Mg	0.65 ± 0.23	0.59 ± 0.25	0.92 ± 0.21^a^	0.65 ± 0.25	0.95 ± 0.21^a^	0.69 ± 0.31
Fe/Zn	0.98 ± 0.31a	0.98 ± 0.30	0.72 ± 0.30^a^	0.95 ± 0.31	0.74 ± 0.31^a^	0.87 ± 0.40
Cu/Fe	0.11 ± 0.05	0.12 ± 0.06	0.11 ± 0.04	0.12 ± 0.06	0.11 ± 0.04	0.13 ± 0.07
Zn/Cu	9.20 ± 3.78a	8.47 ± 3.05	12.5 ± 4.65^a^	9.16 ± 5.08	12.8 ± 5.33^a^	8.45 ± 3.08

Data are means ± SD

*mean* the arithmetic mean, *SD* standard deviation, *N* number of subjects

^a^Sibutramine group *p* < 0.05 vs baseline

^b^Sibutramine group *p* < 0.05 vs 6 weeks

## Discussion

Sibutramine hydrochloride monohydrate is a norepinephrine and serotonin reuptake inhibitor approved for the long-term management of obesity, in conjunction with a reduced calorie diet and behavior modification in patients unable to lose weight by means of diet and lifestyle changes alone. The efficacy of sibutramine has been demonstrated in randomized trials in obese patients [16–18]. Furthermore, its favorable influence on the lipid profile and glucose concentration has been shown in several studies [19–21]. In the study of Tambascia et al. [22], sibutramine demonstrated efficacy in reducing weight, insulin resistance, triglicerides, and uric acid, but not glucose or cholesterol, in non-diabetic women. The results of our study after 6 weeks show a significant decrease only for TC. No change in LDL-C, TG, glucose levels were observed at that time. After 12 weeks, a significant decrease in TC, LDL-C, TG, and glucose was observed.

Some authors suggest that the use of sibutramine could raise blood pressure on account of its peripheral sympathomimetic effect [23]. Contrarily, reports of other authors and the results of our own study show no significant impact of sibutramine on blood pressure or on NO concentration in long-term or short-term treatment [3, 24]. It is suggested that sibutramine elicits a central sympatholytic effect in the brain, counteracting, at least in part, peripheral sympathetic stimulation.

To the best of our knowledge, this is the first clinical study investigating the effect of sibutramine on mineral status in obese women. The results of the experimental studies suggest the potential influence of sibutramine on minerals. Emer et al. [25] found that a relatively high dose of sibutramine resulted in significant increases in Fe and Zn levels in the kidneys and in the adrenal glands in rats. The results of our study show significant changes in mineral levels of obese women treated with sibutramine.

The mechanism may be related to reduced energy intake as an effect of sibutramine action. It has been demonstrated that lower food intake leads to a lower supply of minerals and reduced mineral status in the body. Acuirre et al. [26] reported that a 1,000 kcal/day diet decreases the intake of Fe, Zn, Cu, and Ca by 50 %, 30 %, 40 %, and 9 %, respectively.

This weight-reducing diet led to a reduction of Fe status in obese patients, as indicated by the decrease in serum ferritin. In our study, a significant decrease in ferritin, with a slight (but not significant) reduction in body mass, was also observed, though only in the women taking sibutramine, and not in the group administered the placebo.

However, in the present study, the decreased level of ferritin is connected with increased GSH-Px and SOD level in the blood, which suggests that the change in serum ferritin level is also associated with a change in inflammation in the body of obese women during the sibutramine treatment. Our results may be consistent with the observation of Zafon et al. [27], who claim that ferritin concentration in the obese is due to inflammation, rather than to the Fe status of the body.

The excretion of minerals in the urine of the studied subjects may be a reflection of a change in the mineral status of the body. Changes in the excretion of minerals are not uniform, as shown in Table 3. This is evidenced by the change in molar ratios of elements in the excreted urine. The molar ratios of minerals in the blood also changed. In each tissue of the body, the ion balance is maintained and remains constant. The evaluation of mineral status and mineral metabolism in the body is possible on the basis of the proportions found to hold between different minerals. There are synergistic and antagonistic relationships between the minerals, which directly affect the metabolism of the body. Keeping an appropriate balance between the elements is, in many cases, more important than their normal levels in the tissue. Changes in the ratio between minerals in tissues indicate metabolic disorders in the body and/or interactions between minerals. In the present study, following the sibutramine treatment we observed a decrease in the Ca level (though insignificant) and a higher concentration of Mg in serum. Ca and Mg levels in the body are regulated by a negative-feedback system, and through competition for intestinal absorption and renal reabsorption. Ca and Mg also compete for membrane binding sites within the cell. A lower Ca/Mg ratio in the serum, and higher Ca/Mg ration in urine is probably the result of the disturbed Mg status following sibutramine treatment.

Slightly lower Fe and Zn levels, as well as higher Cu concentrations, were found in the serum after 6 and 12 weeks of the study vs. the baseline. These changes may indicate interactions between the elements due to the use of sibutramine. Known interactions of Ca–Mg and Fe–Zn–Cu can be observed. The higher Cu/Fe ratio and lower Zn/Cu ratio in the serum of patients after treatment may be affected by a disturbed Zn status. Decreasing the Zn level in the organism increases the Cu level, which is a result of the Zn–Cu antagonism.

In other studies, low plasma Zn levels were observed in obese [28], and obese type-2 diabetic individuals [29]. Similarly, rather low plasma Zn level was observed in obese women in the current study. In some studies based on hypocaloric diets and physical activity, weight loss in obese women was associated with increased plasma Zn, and this increase was not a reflection of diet [28]. In this study, we observed an inverse relationship. Moreover, Konukoglua et al. [29] found negative correlations between leptin and Zn in serum among obese diabetic subjects. The results obtained in our study may indicate the effects of sibutramine, but not of weight loss or leptin concentration decrease (not analyzed in this study), on changes in the Zn concentration in the serum of obese women. It has been shown that Zn may contribute to the modulation of serotonin uptake in the brain [30]. Sibutramine is a serotonin uptake inhibitor, and so sibutramine by influencing serotonin metabolism might also indirectly disturb the Zn status of the body. The influence of sibutramine on neurotransmitters may also indirectly affect the level of Cu in the body. It has been found that Cu metabolism is associated with neurological functions (and with the level of serotonin in the brain) [31, 32]. Moreover, Lima et al. did not report any differences in Cu concentration in serum or erythrocytes when comparing obese and non-obese female children [33]. In the present study we observed that, along with the decrease in the concentration of TG and TC, the levels of Fe and Zn lowered significantly, while level of Cu in the serum increased. The results are consistent with the data from our previous study, where obese patients with hypertension and insulin resistance were studied [34].

Treatment with sibutramine resulted in increase in GSH-Px and SOD activity in the serum. GSH-Px is the main enzyme of the enzymatic antioxidant defense system responsible for protecting against increases in ROS production [35]. Hydrogen peroxide, formed by the catalytic reaction of SOD, is both a reactive form of oxygen and a normal cellular metabolite, and it is further detoxified by GSH-Px and catalase [36]. The erythrocyte is a relatively abundant focus of both free-radical-mediated injury by virtue of enhanced endogenous rates of production of ROS and impairment of tissue-free radical defense mechanisms [37, 38]. In addition, GSH is the substrate of GSH-Px, and the cysteine derivative GSH is synthesized in erythrocytes, and glutathione disulfide transported outside the cell to maintain a high GSH/GSSG ratio [35, 38]. Disulfide-exchange reactions occur between protein thiols and low molecular weight disulfides [36]. In our present study, GSH-Px and SOD activities in erythrocytes increased in the treatment groups after the twelfth week. The observed changes may result from an alternation of mineral concentrations during sibutramine treatment. This possible relation was reported by Vivoli et al., who found a positive relationship between serum Cu and Cu–Zn SOD [39]. Moreover Piorunska-Stoltzman et al. [40] reported a positive correlation between Cu and GSH-Px in serum. The increase of erythrocyte GSH-Px and SOD values in patients during sibutramine treatments has also been attributed to the inhibition of free oxygen radicals and lipid peroxidation [41], and the protective effects of sibutramine supplementation on glucose-6-phosphate dehydrogenase activity in erythrocytes.

Our study has some limitations, for example short treatment and the relatively small group of subjects. These limitations result from group selection procedure, requiring women without chronic diseases and not using additional drugs. Moreover, this study was interrupted by the withdrawal of sibutramine license in the EU. Finally, based on these results and the literature data, it is difficult to explain the precise mechanism of the mineral changes in the serum and urine during sibutamine treatment. A better understanding of this mechanism requires further extensive biochemical studies.

## Conclusion

Sibutramine favorably influences the lipid profile and glucose concentration in obese women. Additionally, treatment with sibutramine is associated with increase in GSH-Px and SOD activity. However, 12-week sibutramine therapy decreases Zn and increases Mg concentrations in the serum, leading to mineral imbalances in obese women. The control of minerals levels, particularly of Zn, and the potential need for further supplementation should be considered in patients treated with sibutramine.
